# Effect of CFIm25 knockout on RNA polymerase II transcription

**DOI:** 10.1186/s13104-018-4006-1

**Published:** 2018-12-14

**Authors:** Michael Tellier, Jessica G. Hardy, Chris J. Norbury, Shona Murphy

**Affiliations:** Sir William Dunn School of Pathology, University of Oxford, South Park Roads, Oxford, OX1 3RE UK

**Keywords:** RNA polymerase II, CFIm25, Transcription, Transcription termination, Cleavage and polyadenylation complex

## Abstract

**Objectives:**

Transcription of eukaryotic protein-coding genes by RNA polymerase II (pol II) is a highly regulated process. Most human genes have multiple poly(A) sites, which define different possible mRNA ends, suggesting the existence of mechanisms that regulate which poly(A) site is used. Poly(A) site selection may be mediated by cleavage factor I (CFIm), which is part of the cleavage and polyadenylation (CPA) complex. CFIm comprises CFIm25, CFIm59 and CFim68 subunits. It has been documented that the CPA complex also regulates pol II transcription at the start of genes. We therefore investigated whether CFIm, in addition to its role in poly(A) site selection, is involved in the regulation of pol II transcription.

**Data description:**

We provide genome-wide data of the effect of reducing by 90% expression of the CFIm25 constituent of CFIm, which is involved in pre-mRNA cleavage and polyadenylation, on pol II transcription in human cells. We performed pol II ChIP-seq in the presence or absence of CFIm25 and with or without an inhibitor of the cyclin-dependent kinase (CDK)9, which regulates the entry of pol II into productive elongation.

## Objective

The production of a eukaryotic protein-coding mRNA requires the recognition of a specific poly(A) site sequence at the end of the gene. More than half of all human genes contain more than one poly(A) site with evidence of widespread regulation of gene expression through alternative polyadenylation [1]. Poly(A) site recognition is essential for pre-mRNA cleavage and polyadenylation and requires around 85 proteins [2]. Four multi-subunits complexes are essential for pre-mRNA cleavage: cleavage and polyadenylation specificity factor (CPSF), cleavage stimulation factor (CstF), and cleavage factors I (CFIm) and II (CFIIm) [3]. The role of CFIm in cleavage is still unclear but this complex binds 40–50 nt upstream of the poly(A) site [4]. CFIm comprises two CFIm25 subunits, which binds RNA, and two larger subunits, CFIm59 and CFIm68 [5, 6].

Previous studies have shown that depletion of CFIm25 or CFIm68 promotes proximal poly(A) site usage and thus a shortening of the 3′untranslated region (3′UTR) of many mRNAs [7–9]. This suggests that CFIm normally promotes recognition of the distal poly(A) site. Misregulation of CFIm has been linked to both tumorigenicity of glioblastoma and some neuropsychiatric diseases through changes to mRNAs 3′UTR length [10, 11]. Proteins involved in pre-mRNA cleavage, such as the CPSF complex, regulate pol II activity at the beginning and end of the transcription cycle [12]. To determine if depletion of CFIm25 also affects pol II transcription, we used a CRISPR/Cas9 approach to reduce the expression of CFIm25 and performed pol II ChIP-seq in the absence or presence of a CDK9 inhibitor, which is the kinase regulating pol II entry into productive elongation [13]. Understanding the function of CFIm in pol II transcription could provide insights into transcriptional changes when CFIm is misregulated. Our data should be of interest to the scientific community working on pol II transcription and co-transcriptional processes.

## Data description

HEK293 cells were cultured in Dulbecco’s Modified Eagle’s Medium (DMEM, Sigma) supplemented with 10% fetal bovine serum (FBS, Gibco) and 100 units/ml penicillin + 100 µg/ml streptomycin (Gibco). Two of the three copies of the *CPSF5* gene that encodes CFIm25 were knocked out using CRISPR/Cas9 gene editing and confirmed by sequencing of the edited *CPSF5* locus and by western blotting with an antibody against CFIm25 (NUDT21 10322-1-AP, rabbit polyclonal, ProteinTech), which indicated an approximately 90% reduction in CFIm25 expression in the CFIm25KO cells. HEK293 and CFIm25KO cells were treated prior to ChIP-seq with DMSO or 100 µM DRB (Sigma) for 30 min (Table 1).

**Table 1:** Overview of data files

Label	Name of data file/data set	File types (file extension)	Data repository and identifier (DOI or accession number)
Data file 1	293 DMSO Input	Fastq.gz (raw files), bigwig (processed files)	ENA accession number (fastq.gz): PRJNA490093 GEO accession number (bigwig): GSE119712
Data file 2	293 DMSO Pol II	Fastq.gz (raw files), bigwig (processed files)	ENA accession number (fastq.gz): PRJNA490093 GEO accession number (bigwig): GSE119712
Data file 3	293 DRB Input	Fastq.gz (raw files), bigwig (processed files)	ENA accession number (fastq.gz): PRJNA490093 GEO accession number (bigwig): GSE119712
Data file 4	293 DRB Pol II	Fastq.gz (raw files), bigwig (processed files)	ENA accession number (fastq.gz): PRJNA490093 GEO accession number (bigwig): GSE119712
Data file 5	CFIm25KO DMSO Input	Fastq.gz (raw files), bigwig (processed files)	ENA accession number (fastq.gz): PRJNA490093 GEO accession number (bigwig): GSE119712
Data file 6	CFIm25KO DMSO Pol II	Fastq.gz (raw files), bigwig (processed files)	ENA accession number (fastq.gz): PRJNA490093 GEO accession number (bigwig): GSE119712
Data file 7	CFIm25KO DRB Input	Fastq.gz (raw files), bigwig (processed files)	ENA accession number (fastq.gz): PRJNA490093 GEO accession number (bigwig): GSE119712
Data file 8	CFIm25KO DRB Pol II	Fastq.gz (raw files), bigwig (processed files)	ENA accession number (fastq.gz): PRJNA490093 GEO accession number (bigwig): GSE119712

ChIP was performed as previously described [14]. Briefly, 293 and CFIm25KO cells were crosslinked at room temperature with 1% formaldehyde and quenched with 125 mM glycine for 5 min. Nuclear extracts were sonicated twice for 15 min at high amplitude, 30 s ON/30 s OFF using a Bioruptor (Diagenode). 80 μg of chromatin was incubated overnight at 4 °C with 2 μg of an antibody against IgG (sc-2027, Santa Cruz) as an IP negative control or against pol II (sc-899X, Santa Cruz). After recovery of immune complexes with BSA-saturated protein G Dynabeads and extensive washes, crosslinks were reversed by incubation at 65 °C for 5 h. After ethanol precipitation and proteinase K treatment, DNA was purified using a PCR Purification Kit (Qiagen). ChIP samples were analysed by deep sequencing using Illumina HiSeq 4000 75 bp paired-end reads (Wellcome Trust Centre for Human Genetics, University of Oxford).

To analyse data, adapters were trimmed with Cutadapt v. 1.9.1 [15] with the following constant parameters: --minimum-length 10 –q 15, 10–-max-n 1. Obtained sequences were mapped to the human hg19 reference sequence with Bowtie2 v. 2.2.5 [16]. Unmapped reads were removed with SAMtools v. 1.3.1 [17]. Mapped reads were then de-duplicated using Picard to remove PCR duplicates. Bam files were sorted and indexed with SAMtools. The total number of mapped reads was comprised between 33 and 59 million paired end reads. Bigwig files were created after data normalization to Reads Per Genomic Content (RPGC) by employing deepTools2 v. 2.2.4 [18] bamCoverage tool with the following parameters: -bs 10-normalizeTo1× 2451960000-e–p max.

## Limitations

The effect of CFIm25 KD on pol II transcription is not as strong as the effect observed with knock-down of CFIm68, another member of the CFIm complex [8]. The knockdown efficiency of CFIm25 was about 90%, which may not be sufficient to completely abrogate the role of CFIm25 in regulation of pol II transcription. The ChIP-seq was also performed only once and in only one cell line; HEK293.

## Abbreviations

Pol II: RNA polymerase II
DRB: 5,6-dichlorobenzimidazone-1-β-d-ribofuranoside
ChIP: chromatin immunoprecipitation
RPGC: reads per genomic content
DMEM: Dulbecco’s Modified Eagle’s Medium
FBS: fetal bovine serum
3′UTR: 3′ untranslated region
CDK9: cyclin-dependent kinase 9
CPA: cleavage and polyadenylation complex
CFIm: cleavage factor I
